# Nearly-freestanding supramolecular assembly with tunable structural properties

**DOI:** 10.1038/s41598-023-28865-w

**Published:** 2023-02-05

**Authors:** Tommaso Caruso, Oreste De Luca, Nicola Melfi, Alfonso Policicchio, Michele Pisarra, Nicolas Godbert, Iolinda Aiello, Eugenia Giorno, Daniela Pacilè, Paolo Moras, Fernando Martín, Petra Rudolf, Raffaele Giuseppe Agostino, Marco Papagno

**Affiliations:** 1 Dipartimento di Fisica, Università della Calabria, 87036 Rende (Cs), Italy; 2 Laboratorio di Spettroscopia Avanzata dei Materiali, STAR IR, Via Tito Flavio, Università della Calabria, 87036 Rende (CS), Italy; 3 Zernike Institute for Advanced Materials, University of Groningen, 9747 AG Groningen, The Netherlands; 4 INFN, Sezione LNF, Gruppo Collegato di Cosenza, Cubo 31C, 87036 Rende (CS), Italy; 5 Instituto IMDEA Nanociencia, Calle Faraday 9, 28049 Madrid, Spain; 6 Departamento de Química, Universidad Autónoma de Madrid, Módulo 13, 28049, Madrid Spain; 7 MAT_InLAB (Laboratorio di Materiali Molecolari Inorganici), Centro di Eccellenza CEMIF.CAL, LASCAMM CR-INSTM, Unità INSTM della Calabria, Dipartimento di Chimica e Tecnologie Chimiche, Università della Calabria, 87036 Rende (CS), Italy; 8 LPM-Laboratorio Preparazione Materiali, STAR-Lab, Università della Calabria, Via Tito Flavio, 28049 Rende (CS), Italy; 9 CNR-Nanotec, UoS di Cosenza, Dipartimento di Fisica, Università della Calabria, 87036 Rende (CS), Italy; 10 Istituto di Struttura della Materia-CNR (ISM-CNR), 34149 Trieste, Italy; 11 Condensed Matter Physics Center (IFIMAC), Cantoblanco, 28049 Madrid Spain

**Keywords:** Surfaces, interfaces and thin films, Electronic properties and materials, Molecular self-assembly, Structural properties

## Abstract

The synthesis and design of two-dimensional supramolecular assemblies with specific functionalities is one of the principal goals of the emerging field of molecule-based electronics, which is relevant for many technological applications. Although a large number of molecular assemblies have been already investigated, engineering uniform and highly ordered two-dimensional molecular assemblies is still a challenge. Here we report on a novel approach to prepare wide highly crystalline molecular assemblies with tunable structural properties. We make use of the high-reactivity of the carboxylic acid functional moiety and of the predictable structural features of non-polar alkane chains to synthesize 2D supramolecular assemblies of 4-(decyloxy)benzoic acid (4DBA;C$_{17}$H$_{26}$O$_{3}$) on a Au(111) surface. By means of scanning tunneling microscopy, density functional theory calculations and photoemission spectroscopy, we demonstrate that these molecules form a self-limited highly ordered and defect-free two-dimensional single-layer film of micrometer-size, which exhibits a nearly-freestanding character. We prove that by changing the length of the alkoxy chain it is possible to modify in a controlled way the molecular density of the “floating” overlayer without affecting the molecular assembly. This system is especially suitable for engineering molecular assemblies because it represents one of the few 2D molecular arrays with specific functionality where the structural properties can be tuned in a controlled way, while preserving the molecular pattern.

## Introduction

**Figure 1:**
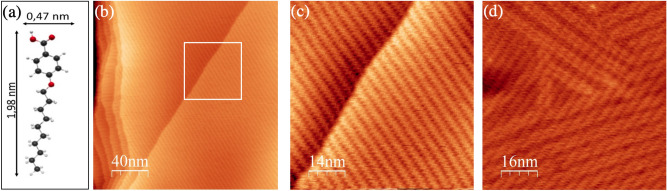
(**a**) Sketch of the 4DBA molecule (carbon atoms are drawn in dark grey, oxygen atoms in red and hydrogen atoms in white). Large scale (**b**) and close-up (**c**) STM images of the molecular assembly of 4DBA on Au(111) (V$_{b}$=1.6 V; I$_{t}$=100 pA; *T*=300 K); (**c**) Represents area marked by the white box (**b**). (**d**) Constant-current STM image of 4DBA/Au(111) (V$_{b}$=1.3 V; I$_{t}$=100 pA; *T*=300 K) displaying both the supramolecular pattern and the herringbone reconstruction.

Complex molecular architectures are usually constructed by connecting molecular building blocks through non-covalent bonding, allowing for a variety of different supramolecular structures and phases to emerge^1–6^. When solid surfaces are used as supports, the molecular self-assembly can be regarded as a two-dimensional (2D) process, and the structural and electronic properties, such as molecular packing, the number and typologies of domains, and the binding energy of the molecular levels, strongly depend on the interplay between the intermolecular forces and the interaction between the adsorbate and the underlying support^7–9^.

A suitable choice of molecular building blocks and supporting substrates would enable, in principle, synthesizing and designing 2D supramolecular assemblies with specific functionalities, disclosing exciting new opportunities in many areas of science and technology^10–13^. Key steps to these accomplishments are (a) to understand how molecules self-arrange, and (b) to master the growth of homogeneous, highly ordered and defect-free films with tunable properties^14,15^.

Small carboxylated molecules present an ideal testing playground for exploring and establishing strategies to design novel molecular assemblies. Owing to their functional groups, these molecules can bind to one another through strong and directional hydrogen bonding. Supported on solid surfaces, these molecular building blocks can self-assemble through electrostatic or van der Waals interaction to form complex 2D architectures^1–6,13,16–28^.

Although hundreds of structures have been realized so far, predicting and designing 2D crystalline materials are still difficult tasks. One remarkable exception are alkyls, which form self-assembled monolayers that can be modulated by varying the chain length^29–32^. In these assemblies the molecules adopt a lamellar structure where the alkyl chains pack side-by-side flat on the surfaces. The specific structure depends on the number of the carbon atoms in the alkyl chain through a mechanism known as “odd-even” effect: Alcohols with an even (odd) number of carbons interact weakly (strongly) with the substrate producing a nearly tilted (orthogonal) alcohol orientation with respect to the lamellae.

The present study takes advantage of the high-reactivity of the carboxylic acid functional moiety and of the predictable structural features of non-polar alkane chains to synthesize nearly-free standing 2D supramolecular assemblies of 4-(decyloxy)benzoic acid (4DBA; C$_{17}$H$_{26}$O$_{3}$) on a Au(111) surface with tunable structural properties. Scanning tunneling microscopy reveals that the self-assembly is self-limited, yielding a highly ordered and defect-free single-layer film of 4DBA molecules with uniform micrometer-sized grains on the noble metal surface. We provide evidence that by varying the length of the alkoxy group it is possible to modify the molecular density without affecting the molecular arrangement, and therefore to tune in a controlled way the structural properties of the supramolecular assembly. By combining high-resolution scanning tunneling microscopy, theoretical modeling and photoemission spectroscopy, we investigated the molecular arrangement on the Au(111) surface and demonstrated the nearly-freestanding character of the 4DBA supramolecular assembly.

## Results and discussion

The 4DBA molecule, sketched in Fig. 1a, is a substituted benzoic acid with a C$_{10}$ alkoxy chain. On the Au(111) surface, the growth of these molecules is self-limited to a single-layer film. A representative scanning tunneling microscopy (STM) image of a 4DBA monolayer on Au(111) is presented in Fig. 1b. On this large scale image, the molecular assembly is visualized as a set of bright parallel stripes on a darker imaged substrate. The assembly covers the wide and atomically flat terraces of the metallic surface, displaying a uniform, defect-free and highly ordered pattern over the whole sample surface investigated by STM (surface area $\sim$ 10 $\upmu$m$^{2}$). In most cases, the molecular stripes display a perfect alignment between the adjacent wide terraces of the metallic substrate, as shown in the magnified view presented in Fig. 1c. Different orientations of the molecular stripes are however observed in those sample regions characterized by several step edges (see left-hand side of Fig. 1b and Fig. S1 of the Supplementary Information).

Scanning tunneling microscopy shows streaks or no image at all for an incomplete single-layer film at all temperatures accessible in our experiment (T > 160 K), indicating that the 4DBA molecules are too mobile for imaging. This behavior is distinctive of weakly interacting overlayers^33,34^.

In Fig. 1d (and Fig. S2a) we present a STM image of the same sample surface collected with a different bias voltage. Here, beneath the molecular network, the Au herringbone reconstruction is also seen as bright lines in the STM image^35–38^. This indicates that the adsorbed layer does not substantially change the surface stress of the substrate at odd with molecular overlayers that chemisorb strongly^37–43^. Furthermore, we note that the orientation of the molecules do not change on the different sides of the elbow position of the herringbone reconstruction (see Fig. S2b of the Supplementary Information). Both results are distinctive of a weakly interacting overlayer.

**Figure 2:**
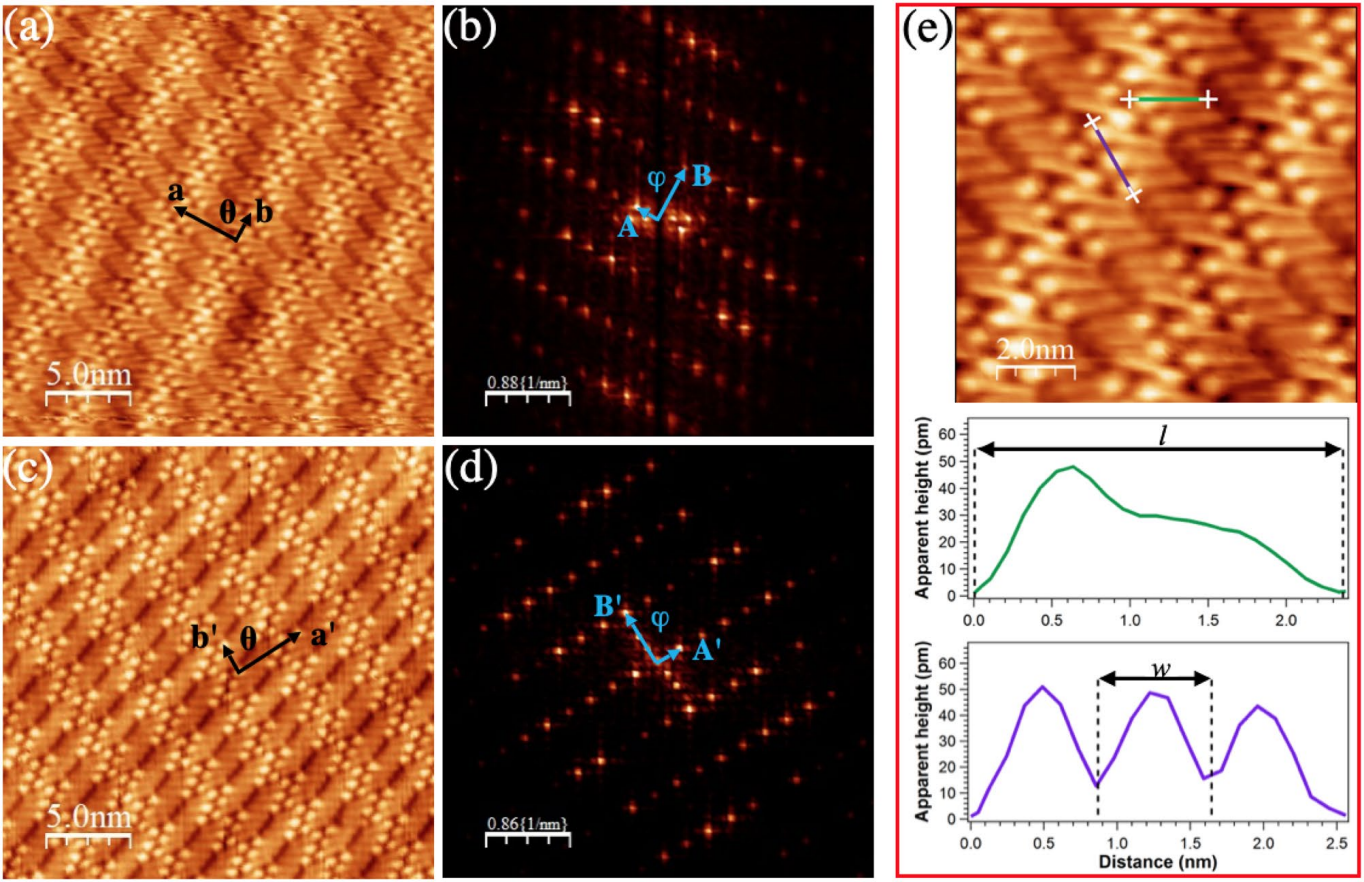
(**a**,**c**) Constant-current STM images (V$_{b}$=1.1 V; I$_{t}$=100 pA; *T*=300 K) displaying two different supramolecular assemblies observed for 4DBA/Au(111); The black arrows represent the unit cell vectors. (**b**) and (**d**) are the Fourier transforms of panels (**a**) and (**c**), respectively; The blue arrows identify the unit cell vectors in the reciprocal space. (**e**) Top panel: STM image of 4DBA/Au(111) (V$_{b}$=1.1 V; I$_{t}$=100 pA; *T*=300 K); Bottom panel: line profiles along the green and blue lines of the top panel marking the length (*l*) and benzoic head width (*w*) of the 4DBA molecule.

High-resolution STM images of single-layer 4DBA unveil the details of the internal molecular structure, orientation, and packing of the ordered adlayer. We found two distinct molecular arrangements, presented in Fig. 2a,c, which both produce stripes on a larger scale. Each molecule is visualized here as a bright spot with a fainter tail; We associate the former to the carboxylic acid group and the latter to the alkoxy chain. Groups of molecules are separated by darker regions (lamellae). Similar molecular arrangements have been observed for monocarboxylic acids deposited on HOPG^21,28^. The Fourier analysis of Fig. 2a,c, displayed in Fig. 2b,d, respectively, proves that, though the arrangements are not equivalent, they share the same unit cell (**a** = 3.9 ± 0.2 nm, **b** = 1.7 ± 0.1 nm, $\Theta$ = 91.5${^\circ }$ ± ${^\circ }$). The unit cell contains 6 molecules and corresponds to a molecular density $\rho _{4DBA}$ = 0.89 nm$^{-2}$.

The line profiles parallel and perpendicular to the molecular axis presented in Fig. 2e provide an estimate of the length (*l* = 1.9 ± 0.1 nm) and benzoic head width (*w* = 0.5 ± 0.1 nm) of the 4DBA molecule, in excellent agreement with the predicted ones^44^, and strongly suggests a parallel adsorption geometry on the metallic surface. This finding is consistent with the planar geometry adopted by monocarboxylic acids on HOPG^21,28^, and by similar molecules like for example benzoic, trimesic or isophthalic acid on Au(111)^20,25,27,32,45^, HOPG^11,29,31^, and benzene on face-centered cubic {111} nobel metal surfaces^7,8,46–50^.

STM investigations on alkyl molecules adsorbed on Au(111)^32^ and HOPG^11,17,19,21,28,29,31^ revealed different 2D molecular patterns depending on the parity (even or odd) of the carbon count in the alkyl chain. Inspired by these results, we explored the possibility to modulate the periodicity of the assembly by changing the alkoxy chain length but always keeping an even number of the carbon atoms.

**Figure 3:**
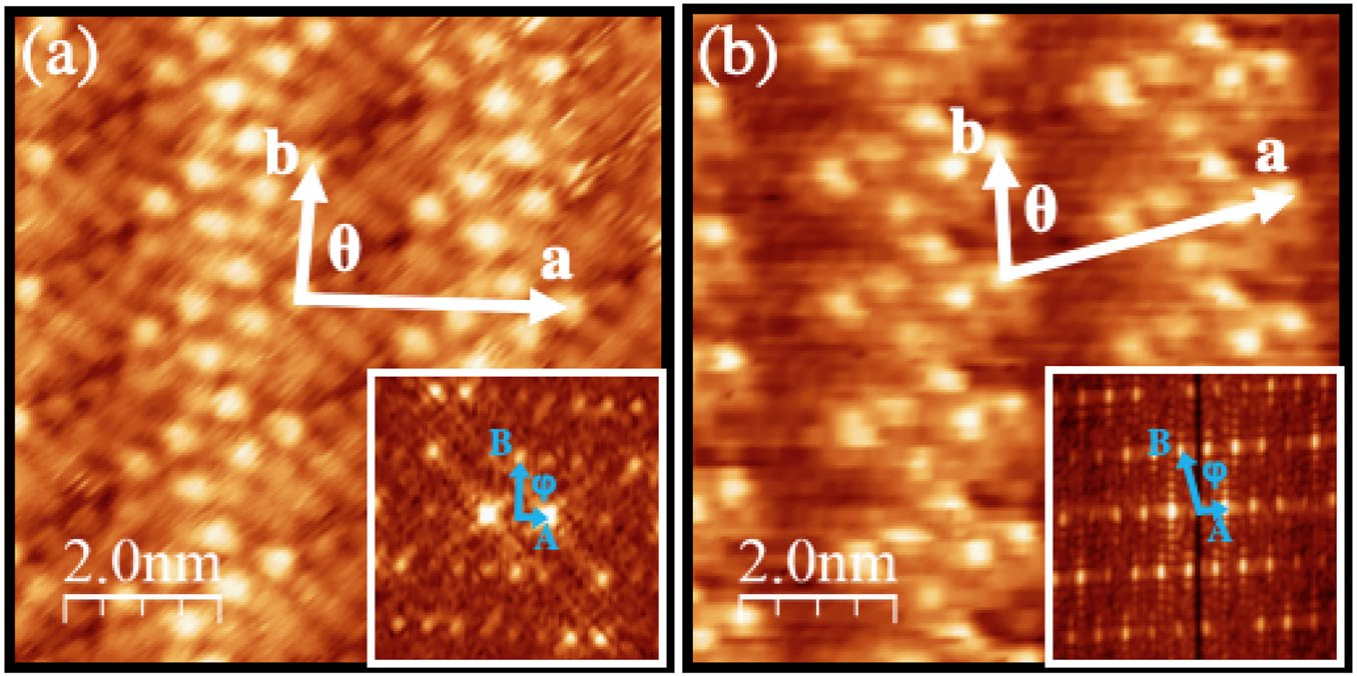
Constant-current STM images of (**a**) 4TBA/Au(111) ( V$_{b}$=0.22 V; I$_{t}$=160 pA; *T*=300 K) and (**b**) 4HBA/Au(111) (V$_{b}$=0.50 V; I$_{t}$=500 pA; T=300 K). White vectors identify the unit cell vectors. The insets of panels (**a**) and (**b**) shows the corresponding Fourier transform; The blue vectors in the insets mark the unit cell vectors in the reciprocal space.

Figure 3a,b shows the STM images collected on the Au(111) surface covered by a single-layer of 4TBA (4-(tetradecyloxy)benzoic acid; C$_{21}$H$_{34}$O$_{3}$) and 4HBA (4-(hexyloxy)benzoic acid; C$_{13}$H$_{18}$O$_{3}$). The former has four more carbon atoms in the chain than 4DBA, while the latter has four less. Collecting STM data on the shorter 4HBA was quite difficult, most likely because of the lower adsorption energy, which reduces the stability of the assembly and hence affects the quality of the image. Both supramolecular assemblies display the same molecular arrangement found for the 4DBA but the unit cells are different (**a** = 3.9 ± 0.2 nm, **b** = 1.8 ± 0.1 nm, $\Theta$ = 93.0${^\circ }$ ± 1.2${^\circ }$ for the 4TBA; **a** = 3.6 ± 0.2 nm, **b** = 1.8 ± 0.1 nm, $\Theta$ = 75.0${^\circ }$ ± 1.2${^\circ }$ for 4HBA), as well as the molecular density, which is  $\sim$10% higher for the 4HBA than for the 4TBA overlayer.

The 4DBA/Au(111) system therefore represents one of the few supramolecular surface assemblies that allows, through a proper choice of the length of the alkoxyl chain, to modulate the structural properties of the ensemble in a controlled way without altering the molecular arrangement.

**Figure 4:**
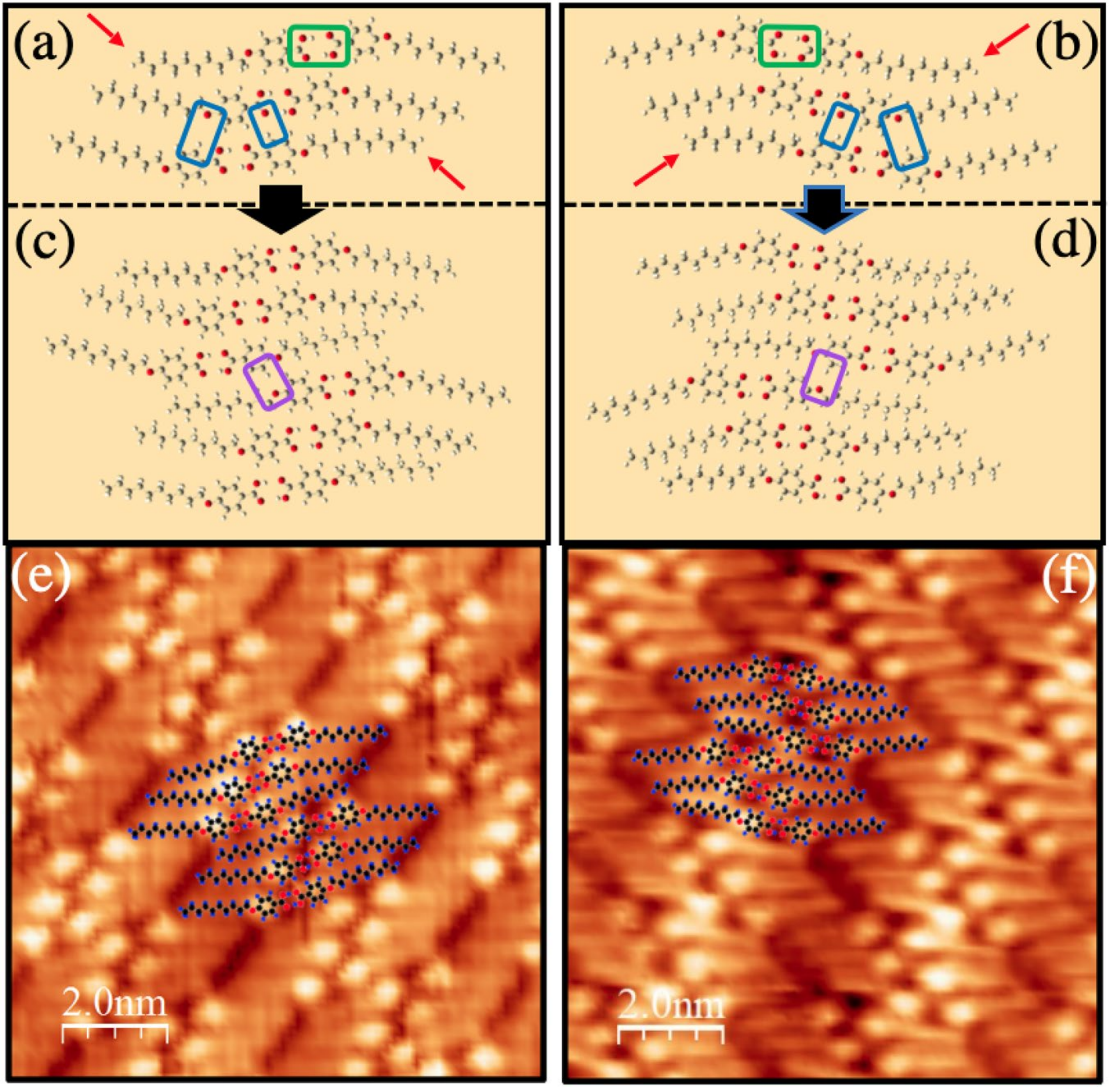
(**a**,**b**) Simulated 4DBA molecular building blocks having opposite supramolecular chirality. The red arrows mark the 4DBA molecules in a different conformation with respect to the others. The green box highlights the double hydrogen bonds within one dimer, whereas the blue boxes mark the electrostatic C–H$\cdots$O interaction between dimers. (**c**,**d**) The optimized supramolecular assemblies from the building blocks presented in panel (**a**) and (**b**), respectively. The purple box encloses the C–H$\cdots$O interaction between adjacent building blocks. (**e**,**f**) The optimized supramolecular structures of panels (**c**,**d**) are overlaid to the STM images of the two experimental assemblies (V$_{b}$=1.1 V; I$_{t}$=100 pA; *T*=300 K).

To gain insight on the observed molecular structure, we investigated the 4DBA assembly by means of first principles atomistic calculations. Inspection of the frontier molecular orbitals (see Fig. S10 in the SI) of 4DBA, 4TBA, and 4HBA shows that the highest occupied molecular orbitals and lowest unoccupied molecular orbitals of these molecules have very similar energies and shapes, demonstrating that the alkyl tail acts as a buffer spacing medium capable of distancing the reactive zones of the molecule in the self-assembly.

STM results suggest that the molecule-substrate interaction plays a minor role in dictating the molecule arrangement. Indeed, test calculations on a simplified system (see Supplementary Information section “2D self assemblies of 4DBA molecules”) show that the molecule-Au(111) interaction is of non-covalent nature. For these reasons, we have analyzed the stability of several self-standing 2D molecular arrangements of 4DBA.

Firstly, we note that, although the 4DBA is an achiral molecule, deposition of a self-assembly of 4DBA molecules on the metal surface can give rise to a surface-induced “supramolecular-chirality”. As shown in detail in section “2D self assemblies of 4DBA molecules” of the Supplementary Information, two equi-energetic 4DBA conformer exists, which only differ in the relative position of the H atom in the carboxylic group with respect to the orientation of the alkyl tail. The 2D self-assembly is realized by two 4DBA forming a dimer through a double hydrogen bond between the carboxylic groups. Such dimers then organize by means of inter-dimer interactions in 2D packed structures (see Fig. 4a,b). We focused on unit cells composed by either 1 or 3 dimers. Our calculations demonstrate that the 3-dimer unit cell arrangements (see Fig. 4a,b) result in a total energy $\sim$ 0.2 eV/molecule lower than that of the 1-dimer unit cell systems. Since the hydrogen atom of the carboxylic functional group is probably free to transfer from one oxygen atom to the other in each molecule and, possibly, within a dimer pair of absorbed molecules, the induced chirality derives from the orientation of the alkoxy chain grafted onto the benzene ring.

Our calculations reveal two equi-energetic 3-dimers arrangements. In both cases, the self-assembly unit cell is made of three 4DBA-dimers bonded by means of double hydrogen bonds (one of the three is highlighted by the green box in Fig. 4a,b) through the carboxilic acid groups with bond length $\sim 2.1$ Å. To minimize the steric repulsion, the alkoxy chains arrange themselves in a fully extended and planar configuration. Each dimer is weakly bonded to the neighboring dimer by weak lateral C–H$\cdots$O interactions^51,52^ (two of them are highlighted by blue boxes in Fig. 4a,b). In the minimum energy configuration, two 4DBA of the molecular building block, highlighted by red arrows in Fig. 4a,b, adopt an inverted conformation with respect to the others. We point out that this is a necessary condition to link adjacent building blocks by means of C–H$\cdots$O interactions (purple box in Fig. 4c,d) and to form a stable 2D system. In this way, the supramolecular assembly optimizes its configuration, minimizing the steric repulsion between the alkoxy chains while increasing the net number of C–H$\cdots$O interactions per molecule.

Interestingly, the unit cells of the two equi-energetic self-assemblies found in the calculations are mirror images, further demonstrating the surface-induced supramolecular chirality^7^. In Fig. 4e,f we overlay the results of our simulations with the STM images of the supramolecular assemblies to emphasize the excellent agreement between the minimum energy arrangement provided by the density functional theory calculations and the experimental STM images.

**Figure 5:**
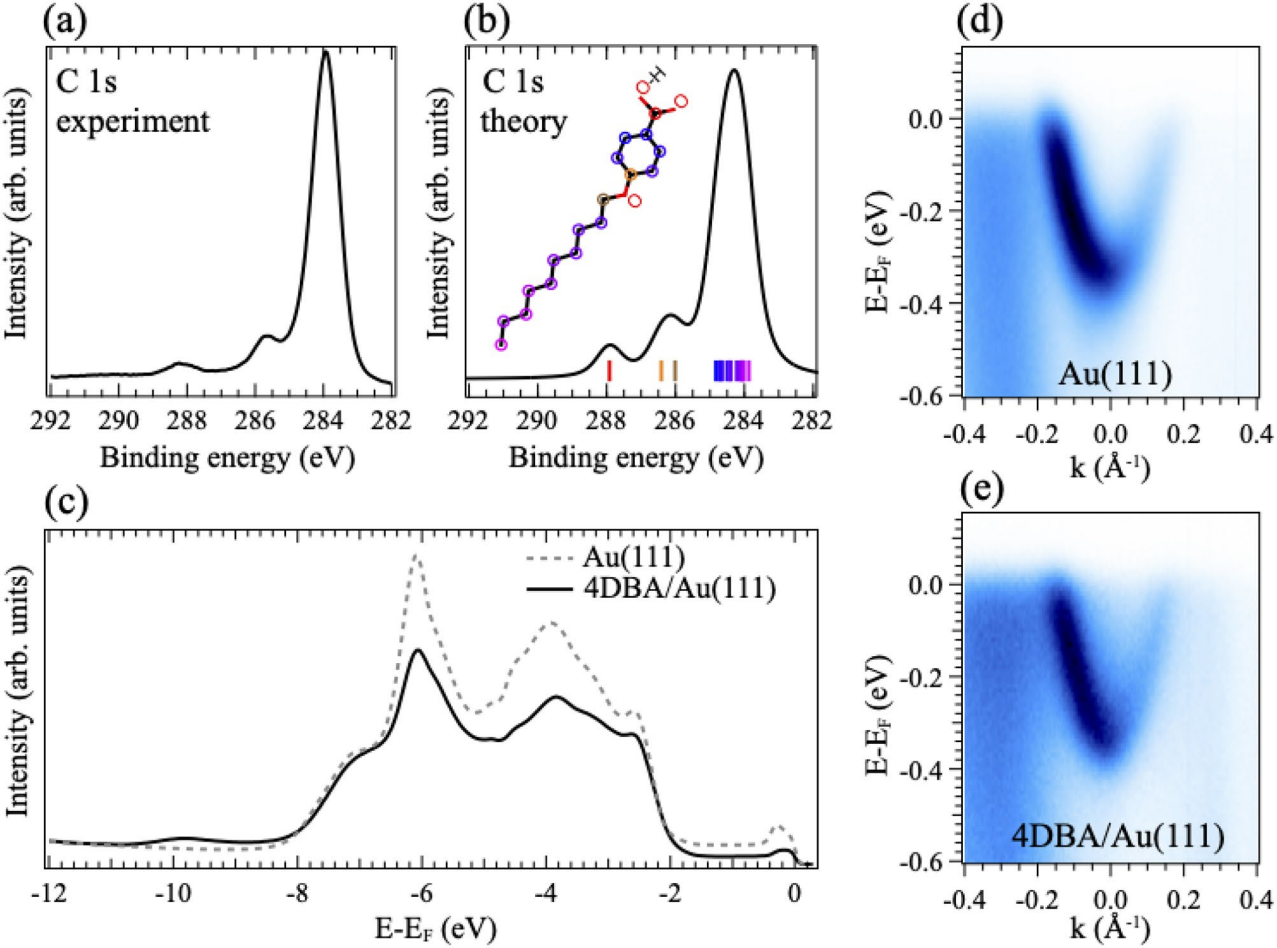
(**a**) Photoemission spectrum of the C 1*s* core level region of 4DBA/Au(111) measured at normal emission with photon energy of 350 eV. (**b**) Simulated C 1*s* photoemission line for 4DBA in gas phase. The energy positions of the 1s state of the different C atoms are marked by different color bars; A sketch of the 4DBA molecule is shown in the inset. A Voigt shape was adopted for each state. (**c**) Valence band for bare Au(111) (dotted gray curve), and 4DBA/Au(111) (full black curve) measured at normal emission with photon energy of 45 eV. ARPES map displaying the Shockley surface state of (**d**) Au(111), and (**e**) 4DBA/Au(111) measured with photon energy of 45 eV.

The electronic structure of 4DBA/Au(111) was investigated by photoemission spectroscopy. The measured spectrum of C 1*s* core level region, presented in Fig. 5a, displays a shape mirroring the presence of differently interacting C atoms. In particular, we observe a main peak at binding energy 284.0 eV and two minor components at 285.7 and 288.1 eV. Similar line-shapes have been recorded for the $\alpha$-form of crystalline *para*-aminobenzoic acid^53^, assemblies of isophthalic acid on Ag(111) and trimesic acids on Cu(111)^22^.

The simulated spectrum of the C 1*s* core level region of a single 4DBA in gas phase, presented in Fig. 5b, is in remarkable agreement with the experimental findings. We included in the inset of Fig. 5b a sketch of the 4DBA molecule with the C atoms marked by different colors. The binding energy of the 1*s* orbital of each C atom is marked by a colored bar on the horizontal axis of the plot. As expected, the C atoms of the COOH group have the highest binding energy, followed by the C atoms bonded to the O atom of the molecular tail. The remainder of the C atoms generates the main peak at the lowest binding energy. The valence band spectrum of clean Au(111), shown in Fig. 5c (gray dotted line), is dominated by the Au 5*d* states in the energy region in between 8 and 2 eV below the Fermi level (E$_F$). Close to E$_F$ a feature due to the Shockley surface state is distinguished, in agreement with previous results^54,55^. After the synthesis of the 4DBA supramolecular assembly, the valence band spectrum (black curve in Fig. 5c) is clearly changed but the high intensity of the Au 5*d* levels prevents a clear identification of the occupied molecular orbitals. The surface state is still present below the supramolecular assembly but its spectral-weight intensity is smaller. This is better seen in the angle-resolved photoemission spectra of Fig. 5d,e presenting the Shockley state of bare Au(111) sample before and after the assembly of the 4DBA overlayer. On Au(111), the Shockley state displays a “parabolic-like” dispersion identical for all in-plane directions with a minimum at the $\bar{\Gamma }$ point^56,57^. Similarly to the herringbone reconstruction, the Shockley state is quite sensitive to the strength of the interaction with the overlayer: For a strong interaction, a substantial shift of the energy minimum toward lower energies and a shorter life-time has been observed, up to a complete disappearance^58–61^. In contrast, the sharpness of the Shockley state along with the unaltered energy minimum observed in Fig. 4f and Fig. S4 supports a negligible 4DBA-Au interaction, in agreement with the STM and DFT findings.

## Conclusion

We characterized the structure and the electronic properties of the 4DBA supermolecular assembly and found that a highly-ordered, uniform and self-limiting defect-free single-layer film covering the whole Au(111) surface is formed. High-resolution STM measurements combined with DFT calculations revealed that the 4DBA molecules self-assemble and interact via a combination of hydrogen bonds and lateral weak C–H$\cdots$O interactions to form a stable molecular 2D network. We prove that by varying the alkoxy chain length of the molecule it is possible to tune the molecular density of the overlayer without affecting the way the molecules arrange. The measured electronic structure supports a nearly-freestanding character of the supramolecular assembly, in agreement with STM and DFT findings. Since an alkoxy chain linked to a molecule with a functional group is the prerequisite for this type of assemblies, systems other than 4DBA should exhibit the same behavior. We therefore believe this work may represent an important step toward engineering molecular devices using supramolecular principles, mostly because the “floating” character of the 4DBA layer implies that the $\pi$ orbitals of the molecules remain practically unsaturated, allowing this system to host foreign atoms and work as a modulating template.

## Methods

STM measurements were performed in ultra-high vacuum (UHV) conditions (base pressure of $5\times 10^{-10}$ mbar) with an Aarhus SPM 150 equipped with KolibriSensor from SPECS operated via Nanonis Control system. STM images were acquired at room temperature (RT) in constant-current mode with a W tip cleaned in UHV by repeated cycles of Ar$^{+}$ sputtering. Tunneling current and voltage are labeled I$_{t}$ and V$_{b}$, respectively. STM measurements were collected both at positive and negative bias voltages resulting in similar results. All STM images were processed using the WSxM software^62^.

Core level and valence band spectra were recorded at RT at the VUV-Photoemission beamline of the Elettra synchrotron radiation facility using a Scienta R4000 electron energy analyzer with a total energy resolution of 30 meV and an angular resolution of 0.1$^{\circ }$.

The Au(111) surface (Phasis, a 200 nm of thick Au(111) layer on mica, 99.99% purity), was cleaned by several cycles of Ar$^{+}$ sputtering (*E* = 1 keV for 20 min) followed by annealing (*T* = 700 K for 20 min). The cleanliness quality of the pristine gold surface was verified with STM or photoemission measurements. 4DBA molecules were deposited at RT on Au(111) by organic molecular beam deposition at a pressure of $3\times 10^{-9}$ mbar using a home-built evaporator. The evaporation temperature was $\sim$ 345 K. Single-layers of molecules were obtained by $\sim$10 sec exposure. A 30 times longer exposure time did not result in multi-layers formation. Annealing the 4DBA/Au at temperatures up to 330 K did not alter the molecular pattern or induced a phase transitions. Annealing at higher temperatures resulted in desorption of the 4DBA molecules.

The electronic structure of both 4DBA conformers were first studied in gas phase using the Quantum Chemistry package Gaussian09^63^. We carried out a full relaxation of all the coordinates, using an unrestricted double-zeta Pople’s 6-31+G(d,p) basis^64–67^ and the B3LYP functional^68^, without imposing any condition on the symmetry of the molecules. These results on the electron structure calculations were also employed to simulate the C 1s and O 1s spectral line shapes.

**Table 1:** Phase transitions temperatures for the synthesized alkoxy-benzoic acid, Cr, Cr’= crystal phases, Sm = smectic, N = nematic, I = isotropic liquid.

Molecule	Phase transitions ($^{\circ }$C)
4-HBA	Cr 73 Cr’ 106 N 152 I heating
	I 151 N 95 Cr’94 Cr cooling
4-DBA	Cr 88 Cr’ 98 Sm 125 N 144 I heating
	I 141 N 123 Sm 94 Cr’76 Cr cooling
4-TBA	Cr 83 Sm 129 I heating
	I 128 N 126 Sm 85 Cr’79 Cr cooling

The molecule self-assembly calculations were carried out using density functional theory within the projector augmented wave (PAW) approach^69^, as implemented in the VASP code^70–72^, using the Perdew-Burke-Ernzerhof (PBE) exchange correlation functional^73^ and the Tkatchenko-Scheffler^74^ corrections, to account for weak dispersion forces. Five different types of periodic self-assemblies were taken into account, containing either two or six 4DBA molecules in the 2D unit cell, using a vacuum region of at least 20 Å in the out-of-plane direction. We adopted a 400 eV plane wave cut-off and a total energy threshold of $10^{-5}$ eV for the self-consistent field calculations. The reciprocal space sampling was carried out using unshifted $6\times 2\times 1$ ($3\times 2\times 1$ for the six-4DBA molecule per unit cell case), $\Gamma$-centered Monkhorst-Pack grids. For each case, several initial conditions (at least three) were tested and the final geometries were obtained relaxing the coordinates of all the atoms until the maximum force was less than 0.01 eV/Å.

The synthesis of the 4-alkoxy-benzoic acid derivatives was performed consistently with the already reported literature^75,76^ through the appropriate modifications reported in Supporting Information (Fig. S5). All three compounds are thermotropic liquid crystals and their thermal behavior was analyzed by differential scanning calorimetry (DSC) and the textures of the mesophases were observed under polarized optical microscope (POM) equipped with a heating stage. Thermotropic behavior of alkoxybenzoic acids is well known and is attributed to the formation of dimers through the carboxylic head-group. The DSC curves and POM micrographs are reported in Supporting Information (Figs. S6–S9). All compounds display polymorphism in their solid state, most likely attributable to a change of conformation of the alkyl chains in the solid state. While 4HBA presents only a nematic mesophase, the longest alkyl chain lengths alkoxybenzoic acids (4DBA and 4TBA) display both smectic and nematic mesophases. However, the temperature range of the monotropic nematic phase displayed by 4TBA is rather reduced (127–129 $^{\circ }$C) but well distinguishable due to the observation of a characteristic fingerprint texture displayed on slow cooling from the isotropic temperature (129 $^{\circ }$C), just before entering into its smectic mesophase at 127 $^{\circ }$C (see Supporting Information for relative POM micrographs). The observation for (alkoxy)benzoic acids of this fingerprint texture, which is usually observed for cholesteric liquid crystals (Chiral nematics) has been postulated to be due to the a chiral-like behaviour of opened dimers. This chiral-like behavior disappears upon further cooling with the concomitant closure of the dimers^77^. The organization of the mesophases for all three compounds are reported in Table 1, and are consistent with results obtained from reported analogues of different or similar alkyl chain lengths^77,78^.

## Supplementary Information

Supplementary Information.

## Data Availability

The data that support the findings of this study are available from the corresponding authors upon request.
