# Supplementary material for: Nearly-freestanding supramolecular assembly with tunable structural properties
Source: Sci Rep. 2023 Feb 5;13:2068. doi: 10.1038/s41598-023-28865-w (PMC9899781; doi:10.1038/s41598-023-28865-w)
Supplement: Supplementary file 1 — Supplementary Information. [file 41598_2023_28865_MOESM1_ESM.pdf]

Supplementary Information
Nearly-Freestanding Supramolecular Assembly with Tunable Structural Properties

Tommaso Caruso[1,2,+], Oreste De Luca[2,3,+], Nicola Melfi[1], Alfonso Policicchio[1,2], Michele Pisarra[4,5,6], Nicolas Godbert[7,8], Iolinda Aiello[7,8,9], Eugenia Giorno[7,8], Daniela Pacilè[1], Paolo Moras[10], Fernando Martín[5,6,11], Petra Rudolf[3], Raffaele Giuseppe Agostino[1,2], and Marco Papagno[1,2,*]

[1]Dipartimento di Fisica, Università della Calabria, 87036, Rende (Cs), Italy
[2]Laboratorio di Spettroscopia Avanzata dei Materiali, STAR IR, Via Tito Flavio, Università della Calabria, 87036, Rende (CS), Italy
[3]Zernike Institute for Advanced Materials, University of Groningen, 9747 AG, Groningen, Netherlands
[4]INFN, sezione LNF, Gruppo collegato di Cosenza, Cubo 31C, 87036, Rende (CS), Italy
[5]Instituto IMDEA Nanociencia, Calle Faraday 9, 28049, Madrid, Spain
[6]Departamento de Química, Módulo 13, Universidad Autónoma de Madrid, 28049, Madrid, Spain
[7]MAT_InLAB (Laboratorio di Materiali Molecolari Inorganici), Centro di Eccellenza CEMIF.CAL, LASCAMM CR-INSTM, Unità INSTM della Calabria, Dipartimento di Chimica e Tecnologie Chimiche, Università della Calabria, 87036, Rende (CS), Italy
[8]LPM-Laboratorio Preparazione Materiali, STAR-Lab, Via Tito Flavio, Università della Calabria, 87036, Rende (CS), Italy
[9]CNR-Nanotec, UoS di Cosenza, Dipartimento di Fisica, Università della Calabria, 87036, Rende (CS), Italy
[10]Istituto di Struttura della Materia-CNR (ISM-CNR), 34149, Trieste, Italy
[11]Condensed Matter Physics Center (IFIMAC), Cantoblanco, 28049 Madrid, Spain
[*]corresponding. marco.papagno@fis.unical.it
[+]these authors contributed equally to this work

ABSTRACT

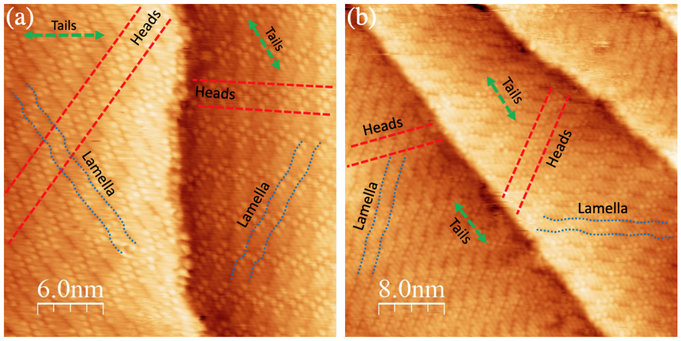

Figure S1. (a)-(b) Constant-current STM images displaying different terraces of the Au(111) surface covered by 1 ML 4DBA (V_b=1.0 V; I_t=100 pA; T=300 K). In both panels, double green arrows are drawn parallel to the alkyl chains, red broken lines identify the direction of one unit cell vector, blue dotted lines mark the lamellae.

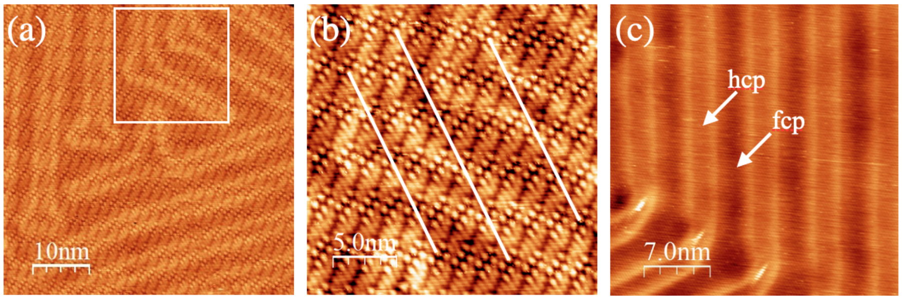

Figure S2. High-resolution STM image of (a) 4DBA/Au(111) (V_b=1.30 V; I_t=100 pA; T=300 K). (b) Magnified view of the STM image enclosed by the white square in panel (a). The white lines trace the molecules orientation within the supramolecular assembly. The angle difference between the lines is smaller than 0.5°. (b) High-resolution STM image of the Au(111) surface (V_b=150 mV; I_t=1.0 nA; T=300 K).

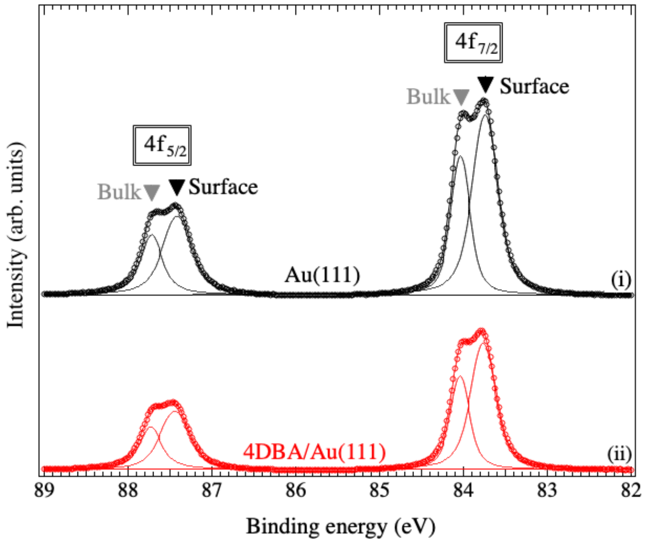

Figure S3. Photoemission spectra of the Au 4f core level region of (i) bare Au(111) and (ii) 4DBA/Au(111). The spectra were collected with a photon energy of 135 eV at normal emission and fitted with Voigt profiles after the subtraction of a Shirley background.

Scanning tunneling microscopy supporting data

Fig. S1 presents constant-current STM images of the supramolecular 4DBA assembly extending over two adjacent Au(111) terraces. The whole assembly can rotate when crossing a step edge while preserving the same molecular arrangement, as shown in panel (a). In some other cases, as in panel (b), the assembly displays an inverse chirality.

Fig. S2(a) displays a high-resolution STM image of 4DBA/Au(111) whereas Fig. S2(b) show the magnified view enclosed by the white square. The orientation of the molecules (highlighted by white lines) is the same (within $< 0.5°$) irrespective of the rotation of the herringbone reconstruction underneath. Fig. S2(c) shows the herringbone reconstruction of Au(111); The bright lines are due to the soliton walls running along the $\left[\bar{1}10\right]$ directions, dividing face-centered-cubic (fcc) and hexagonal closed-packed (hcp) surface sites, as indicated by the white arrows in the figure.

1 Photoemission spectroscopy supporting data

Fig. S3 shows the Au $4f_{7/2}$ and $4f_{5/2}$ core level photoemission spectra of bare Au(111) surface and the same surface covered with 4DBA both displaying components arising from the bulk (at binding energies of 84.04 and 87.72 eV) and from the surface (at 83.74 and 87.42 eV), in agreement with previous works.[1–3] The 4DBA supramolecular assembly barely affects the surface components: from the fits we deduce a decrease of the photoemission spectral-weight of about 20% and a variation by less than

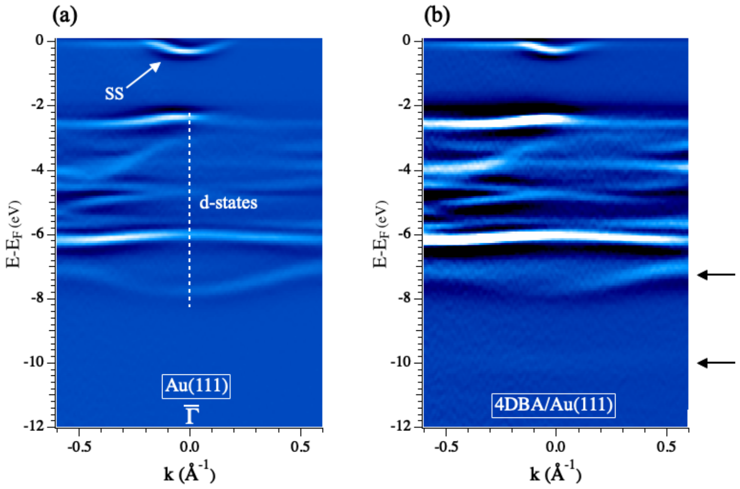

Figure S4. Second derivative along the energy axis of the energy and momentum dispersions for (a) Au(111) and (b) 4DBA/Au(111). In (a) the arrow marks the Shockley surface state and the white dotted line the *d*-states energy range. $\bar{\Gamma}$ marks the center of the surface Brillouin zone. Black arrows in (b) highlight 4DBA induced molecular states. The angle-resolved photoemission data were recorded with a photon energy of $h\nu = 45$ eV at 300 K.

5% of the energy and the width of the $4f$ peaks.

In Fig. S4(a) the second derivative of the photoemission intensity along the energy axis $\frac{\partial^2 I(E,\mathbf{k})}{\partial E^2}$ is presented for Au(111), in agreement with the literature.[4] The observed features are due to the *d*-states, between -8 and -2 eV (white dotted line), and to the Shockley surface state (white arrow), close to zero energy at $\bar{\Gamma}$. The synthesis of the supramolecular assembly in Fig. S4(b) induces at least two electronic states (highlighted by black arrows) derived from the molecular orbitals, which, within the experimental resolution, do not show clear hybridization with the substrate bands, at odds with other nearly-freestanding overlayers.[3,5,6]

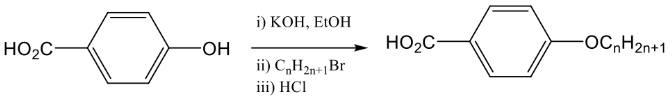

Figure S5. Schematic representation of the synthesis of the 4 alkoxybenzoic acids.

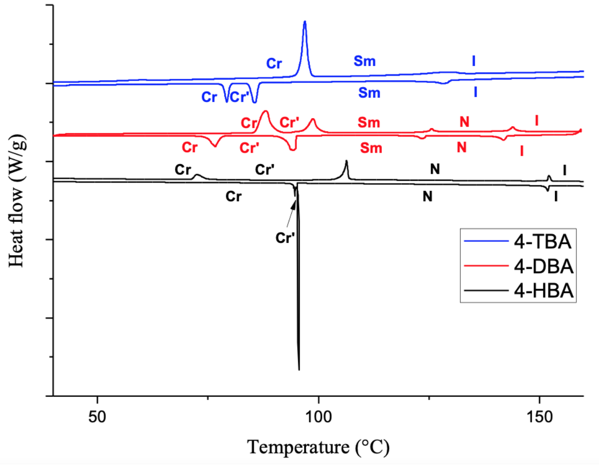

Figure S6. DSC curves obtained for 4-HBA black line, 4-DCA red line and 4-TBA blue line. Cr, Cr'=crystal phases, Sm=smectic, N=nematic, I=isotropic liquid.

2 Synthesis of the molecules

2.1 General procedure for the synthesis of 4 alkoxybenzoic acids (4-HBA, 4-DBA and 4-TBA)

The 4-hydroxybenzoic acid (10 mmol) (Fig. S5) was dissolved in ethanol (50 mL) and 30 mmol of KOH, the resulting reaction mixture was stirred for further ten minutes prior the addition of 20 mmol of the corresponding 1-bromoalkane. The resulting mixture was finally stirred at reflux for 7 h. After being cooled to room temperature, the mixture was acidified with 1 M HCl(aq) until pH=7. The resulting precipitate was isolated by filtration, washed with ethanol and the white-off solid was recrystallized from hexane to yield pure white crystals of the corresponding alkylated benzoic acids in ca. 65-72% yields.

4-hexyloxybenzoicacid (4-HBA): Yield: 70% (3.23 g). M.p.: 106°C. ^{1}H NMR (300 MHz, $CDCl_3$) δ: 0.90 (*t*, 3H, 6.9 Hz), 1.27 (*m*, 7H), 1.77 (*m*, 2H, 6.1 Hz), 3.97 (*t*, 2H), 6.96 (*d*, 2H, 7.2 Hz), 6.92 (*d*, 2H, 8.2 Hz), 7.90 (*d*, 2H, 7.9 Hz).
4-decyloxybenzoicacid (4-DBA): Yield: 65% (2.38 g). M.p.: 98°C. ^{1}H NMR (300 MHz, $CDCl_3$) δ: 0.86 (*t*, 3H, 7.0 Hz), 1.27 (*m*, 12H), 1.833 (*t*, 2H, 6.8 Hz), 3.97 (*t*, 2H, 6.6 Hz), 6.94 (*d*, 2H, 7.1 Hz), 6.92 (*d*, 2H, 6.3 Hz), 8.51 (*d*, 2H, 5.7 Hz).
4-tetradecyloxybenzoicacid (4-TBA): Yield: 72% (2.08 g). M.p.: 83°C. ^{1}H NMR (300 MHz, $CDCl_3$) δ: 0.95 (*t*, 3H, 6.8 Hz), 1.59 (*m*, 22H), 1.86 (*t*, 2H, 6.2 Hz), 4.02 (*t*, 2H, 5.5 Hz), 6.95 (*d*, 2H, 7.1 Hz), 8.06 (*d*, 2H, 6.9 Hz).

2.2 Characterization of the 4-alkoxy-benzoic acid derivatives by differential scanning calorimetry (DSC)

All three synthesized compounds are thermotropic liquid crystals and their thermal behavior was analyzed by differential scanning calorimetry (DSC). Measurements were carried out using TA DSC Q2000 instrument with nitrogen as a purge gas, at a flow rate of 50 ml·min^{-1}. Accurately weighed samples (1.5-2 mg of compounds) were sealed in non-hermetic aluminium pans and heated from 40 to 160 °C with a heating rate of 10 °C/min. Note that all compounds display polymorphism in their solid state (Fig. S6), most likely attributable to a change of conformation of the alkyl chains.

Mesophase organizations was assigned through the observation of a small amount of sample sandwiched between two microscope glass slides and placed under a polarized microscope equipped with a heating stage (POM). The respective micrographs for 4HBA, 4DBA, and 4TBA are reported in Fig. S7-S9.

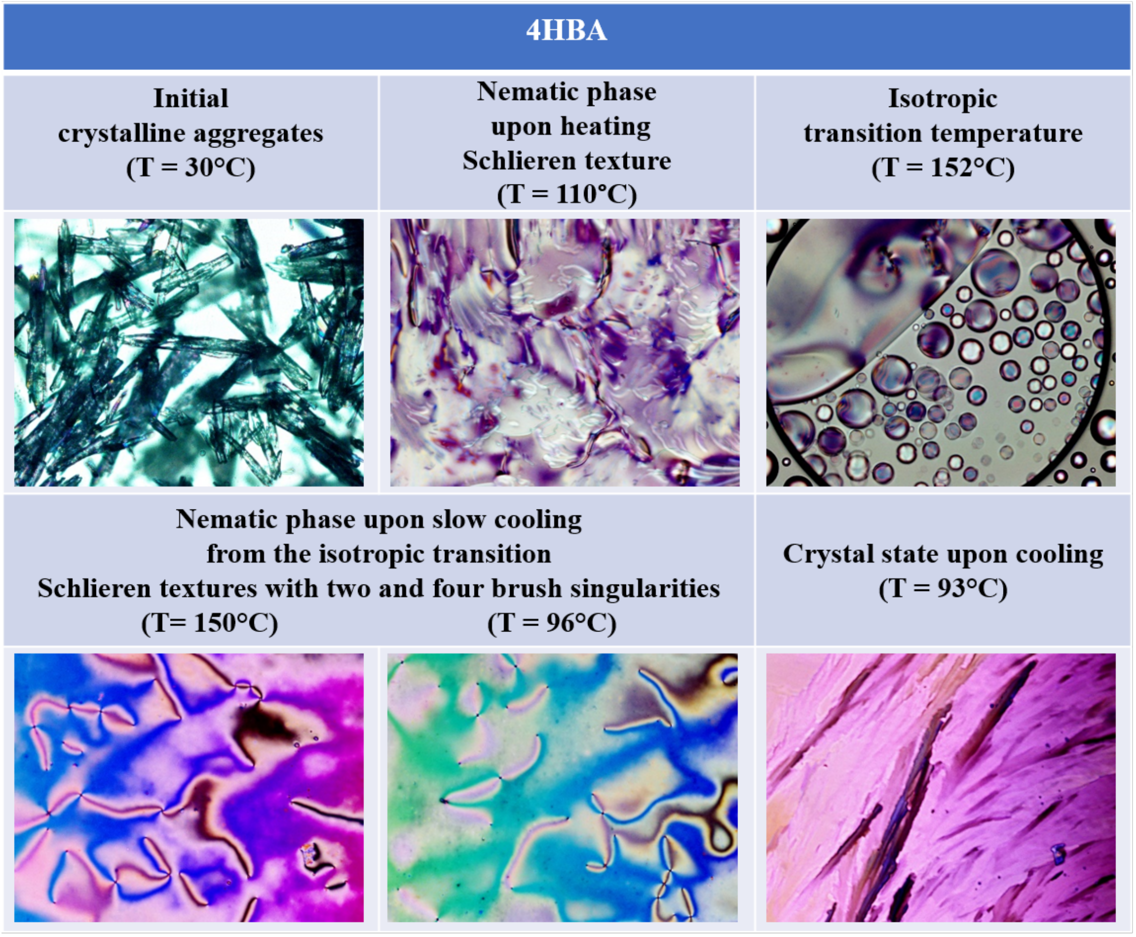

Figure S7. Micrographs of 4HBA observed under POM.

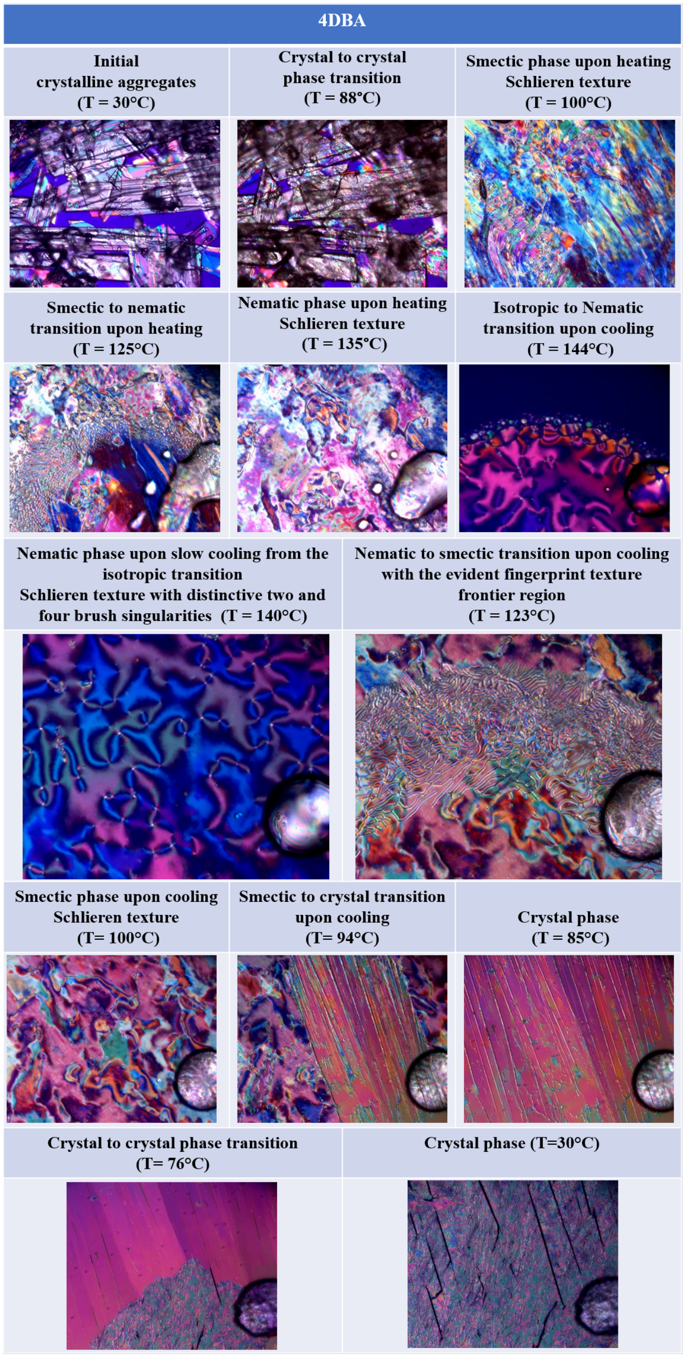

Figure S8. Micrographs of 4DBA observed under POM.

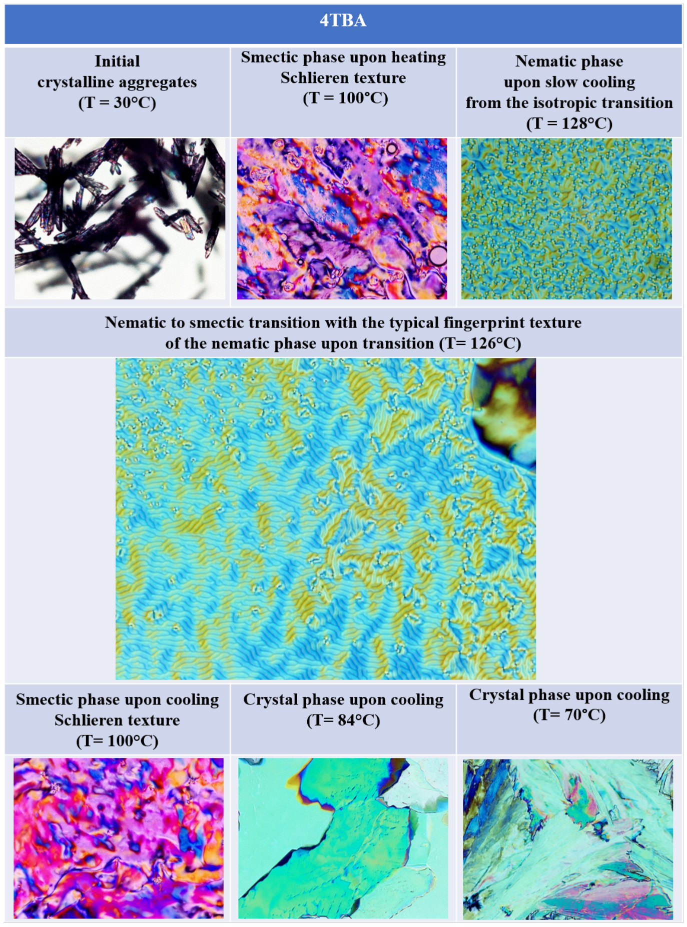

Figure S9. Micrographs of 4TBA observed under POM.

3 DFT and atomistic calculations

3.1 Frontier molecular orbitals for the 4DBA, 4HBA, and 4TBA molecules

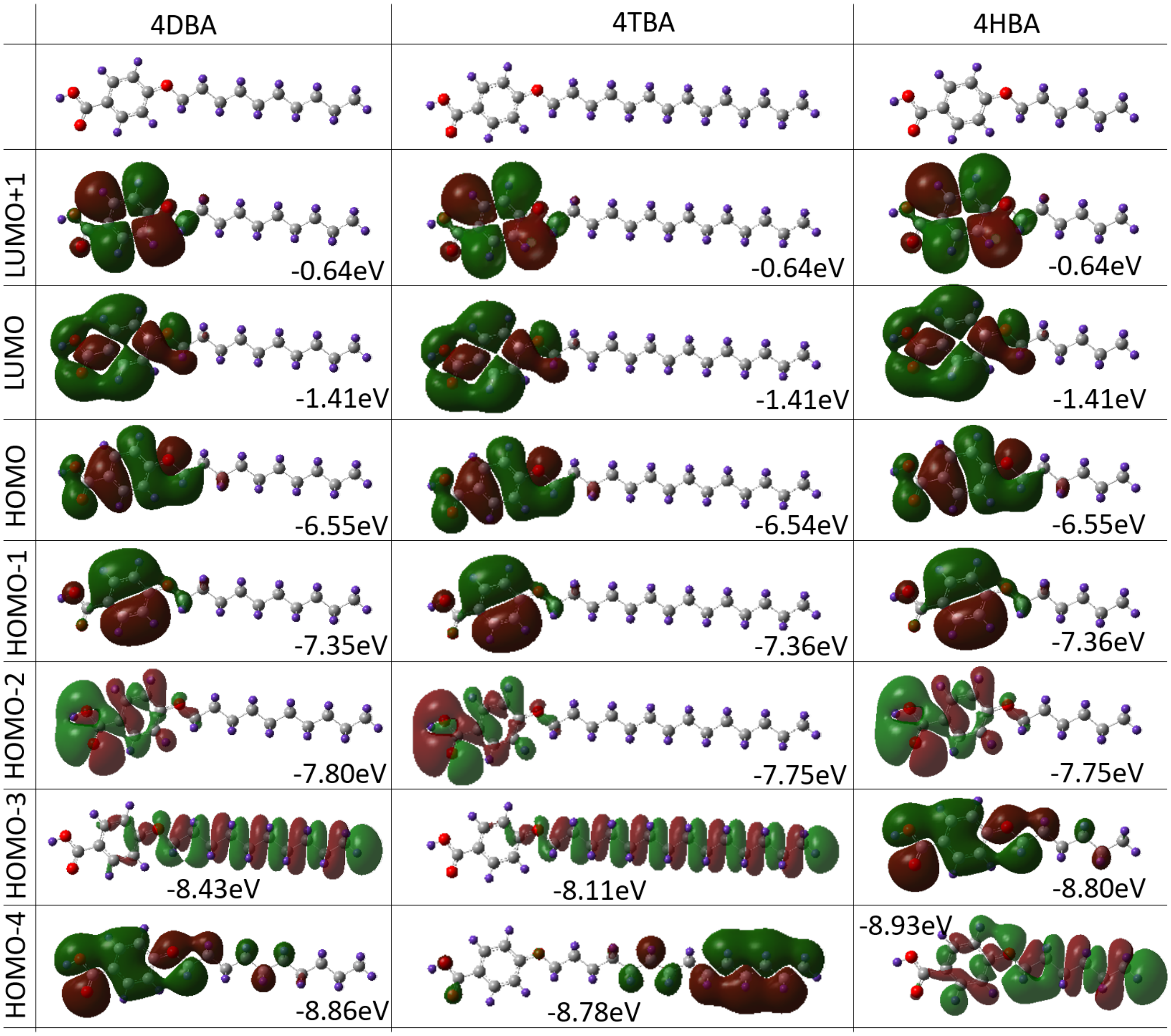

Figure S10. Frontier molecular orbitals of the 4DBA (first column), 4TBA (second column), and 4HBA (third column), as calculated in gas phase: The red and green color mark the 0.005 and -0.005 isosurfaces, respectively. In each case the energy of the molecular orbital is reported as inset. The images have been produced using Gaussview6.1.1.

Fig. S10 demonstrates that the investigated molecules have very similar highest occupied molecular orbitals (HOMOs) and lowest unoccupied molecular orbitals (LUMOs). They are mainly located in the carboxylic acid group and are practically unaffected by the length of the alkoxy chain. This fact also implies that the most reactive part of the molecule is the carboxylic acid group whereas the alkoxy chain is relatively inert. Note that the character of the HOMO-3 and HOMO-4 orbitals in 4HBA are exchanged.

3.2 Molecule-Au(111) interaction

In order to estimate the 4DBA-Au(111) interaction and to understand the influence that the Au(111) surface has in driving the order of the 4DBA self-assembly we have set up a simplified model, which has allowed us to carry out a thorough adsorption study keeping the computational costs to a manageable level. For this study we chose the small 4BBA molecule (4-(bioxy)benzoic acid; $C_9H_{10}O_3$), which has the same "head" as the 4DBA molecule and the shortest possible tail (with an even number of C atoms). We have studied the adsorption of this molecule over a 6-layer Au(111) slab constructing a (6×4) in-plane Au supercell. Given the short tail of the molecule, this supercell is large enough to avoid intermolecule interactions

between the periodic replicas, inherent to the periodic DFT approach. In this way, we can study the interaction of an isolated molecule of the 4*BA family with the gold substrate. We have carried out 10 different geometry optimization varying the relative initial position and orientation of the 4BBA molecule on the gold surface. All the calculations resulted in the adsorption of the molecule in non-equivalent positions with very similar adsorption energies of $E_{ads} = 1.28 \pm 0.03$ eV. The relatively high adsorption energy should not mislead on the molecule-Au interaction nature, which has a dispersing character. This fact is the consequence of the large interaction area and has been corroborated by repeating the optimization without including the van der Waals TS correction. In this test calculations the molecule desorbed from the surface. Furthermore, we found that the molecule lies flat with the benzene ring parallel to the Au surface, with an average distance between the molecule and the topmost Au layer of ~ 3.35 Å, which is a typical marker of non-covalent molecule-surface binding. Finally, we found negligible energy differences between different relative molecule-surface positions, indicating that the molecule is bound to the gold surface by non-covalent interactions and hence can "fluctuate freely" on the surface. Hence, we can conclude that the Au substrate has a negligible role in dictating the geometry of the molecule self-assembly.

3.3 2D self-assemblies of 4DBA molecules

We start pointing out that two 4DBA molecules can form a dimer through a double hydrogen bond between the COOH terminations. Our gas phase calculations find that the dimer formation is energetically favorable with a ~ 0.9 eV binding energy, in fairly good agreement with the double hydrogen bond of acetic acid[7]. We point out that the dimer is the minimum unit to be considered for the formation of a self-assembly. Guided by the STM findings, we have constructed planar arrangements of 4DBA molecules, by laterally repeating 4DBA dimers. Since the 4DBA molecule has 2 conformers (called for simplicity A and B, see Fig. S11) several non-equivalent unit cell can be constructed depending on both the type of dimer and the lateral arrangement of the dimers. In this study we focused on 1-dimer (2 molecules) and 3-dimers (6 molecules) unit cells. In Fig. S11 we report 4 different self-assemblies of 4DBA molecules. In the central row we provide two different arrangements in which the unit cell is made of one dimer (with two different dimers). On the other hand, in the bottom row of Fig. S11 we report the two non-equivalent self-assemblies with minimum energy (see Fig. 4 in the main text), characterized by a unit cell composed of 6 molecules (3 dimers). These latter configurations have a ~ 0.2 eV/molecule lower total energy as compared to the one dimer arrangements. In Fig. S11 the two non-equivalent self-assemblies are depicted in a way that they appear clearly as mirror images, which differ for the type of conformer entering the unit cell, as indicated in the label. It is worth pointing out that as far as self-standing 2D self-assemblies of 4DBA molecules are considered, the two images can be modified so that they appear very similar by changing the viewpoint (above or below) in the rendering. When the self-assemblies are adsorbed on a surface, on the other hand, the above/below arbitrary choice is no longer possible. Hence the observed non-equivalent self-assemblies are a true manifestation of the surface induced pseudo-chirality.

References

1. Heister, K., Zharnikov, M., Grunze, M. & Johansson, L. S. O. Adsorption of alkanethiols and biphenylthiols on au and ag substrates: A high-resolution x-ray photoelectron spectroscopy study. *The J. Phys. Chem. B* **105**, 4058–4061, DOI: 10.1021/jp010127q (2001). https://doi.org/10.1021/jp010127q.
2. Jia, J. *et al.* Selenium adsorption on au(111) and ag(111) surfaces: Adsorbed selenium and selenide films. *The J. Phys. Chem. C* **117**, 9835–9842, DOI: 10.1021/jp4007203 (2013). https://doi.org/10.1021/jp4007203.
3. Dendzik, M. *et al.* Growth and electronic structure of epitaxial single-layer ws_2 on au(111). *Phys. Rev. B* **92**, 245442, DOI: 10.1103/PhysRevB.92.245442 (2015).
4. Sheverdyaeva, P. M. *et al.* Energy-momentum mapping of d-derived au(111) states in a thin film. *Phys. Rev. B* **93**, 035113, DOI: 10.1103/PhysRevB.93.035113 (2016).
5. Starodub, E. *et al.* In-plane orientation effects on the electronic structure, stability, and raman scattering of monolayer graphene on ir(111). *Phys. Rev. B* **83**, 125428, DOI: 10.1103/PhysRevB.83.125428 (2011).
6. Papagno, M. *et al.* Hybridization of graphene and a ag monolayer supported on re(0001). *Phys. Rev. B* **88**, 235430, DOI: 10.1103/PhysRevB.88.235430 (2013).
7. Zhang, M., Chen, L., Yang, H. & Ma, J. Theoretical study of acetic acid association based on hydrogen bonding mechanism. *The J. Phys. Chem. A* **121**, 4560–4568, DOI: 10.1021/acs.jpca.7b03324 (2017). PMID: 28548858, https://doi.org/10.1021/acs.jpca.7b03324.

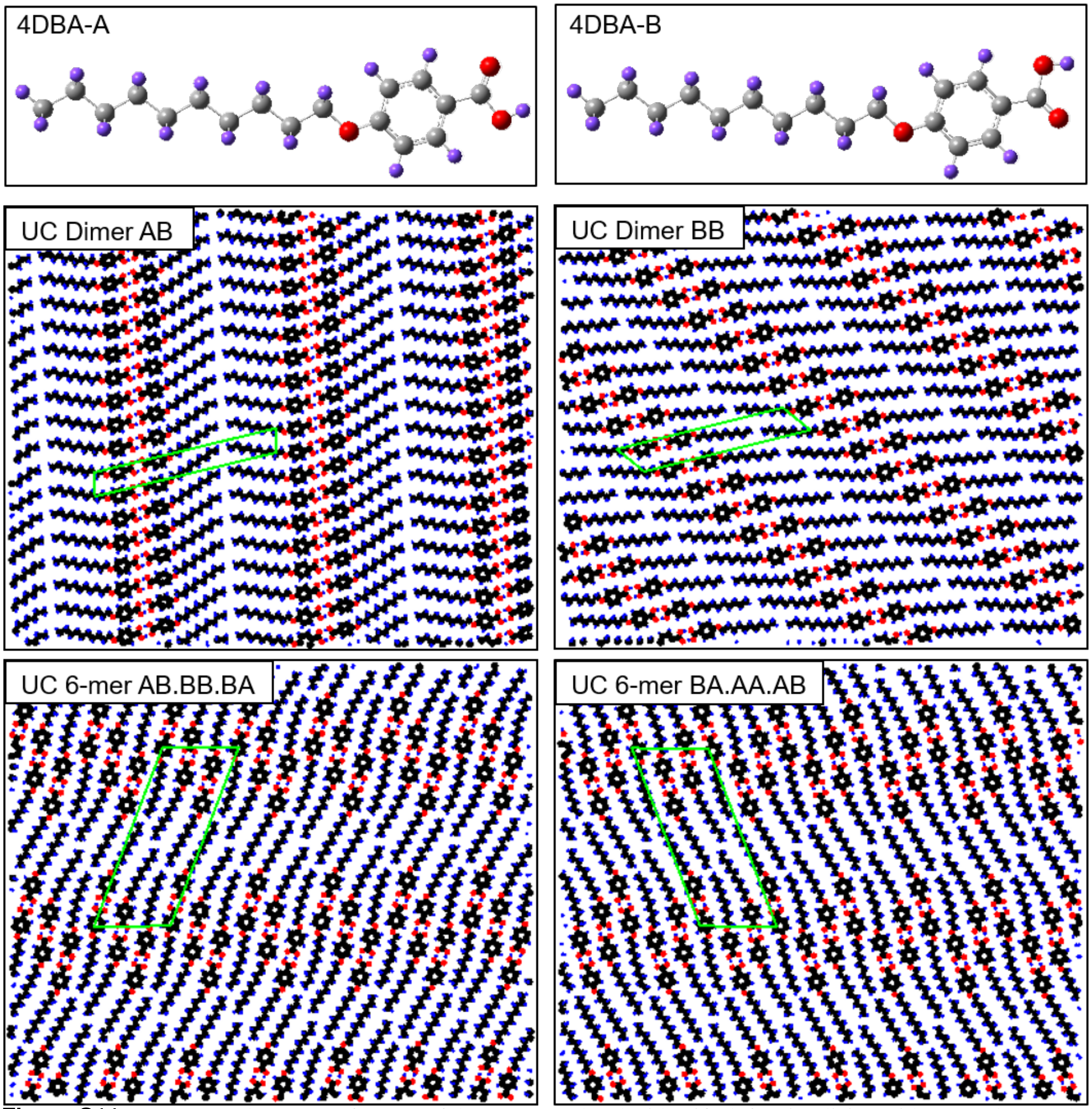

Figure S11. Top Row: The two conformers of the 4DBA molecule, identified, for simplicity, with "A" and "B". The two geometries differ for relative orientation of the COOH head and are transformed one into the other by a 180° rotation around the C-COOH single bond in the head or the C-O single bond in the tail. Middle Row: Optimized geometry of two 2D self-assemblies characterized by a unit cell made of a single 4DBA dimer; The dimer entering the unit cell is different in the two cases, indicated in the label; The green line marks a possible choice of the in-plane unit cell. Bottom Row: Optimized position of two 2D self-assemblies characterized by a unit cell made of three 4DBA dimers; The green line marks a possible choice of the in-plane unit cell.
